# 2023 ISCB Overton Prize: Jingyi Jessica Li

**DOI:** 10.1093/bioinformatics/btad307

**Published:** 2023-06-30

**Authors:** Christiana N Fogg, Diane E Kovats, Martin Vingron

**Affiliations:** Kensington, MD, United States; International Society for Computational Biology (ISCB), 525K East Market Street, RM 330, Leesburg, VA 20176, United States; International Society for Computational Biology (ISCB), 525K East Market Street, RM 330, Leesburg, VA 20176, United States; Max-Planck-Institute for Molecular Genetics, Computational Molecular Biology, Ihnestr. 73, Berlin 14195 D, Germany

The ISCB Overton Prize recognizes early or mid-career scientists as emerging leaders in computational biology or bioinformatics who have made significant research, education, and service contributions to the field. In 2001, the Overton Prize was established to honor the untimely loss of G. Christian Overton, a leader in the field of bioinformatics and a founding member of the ISCB Board of Directors. The 2023 Overton Prize winner is Dr. Jingyi Jessica Li, a Professor in the Department of Statistics (primary), Department of Human Genetics and Department of Biomathematics (secondary) at the University of California, Los Angeles (UCLA). She will receive her award and give a keynote talk at the Joint ISMB/ECCB conference in Lyon, France, this July.


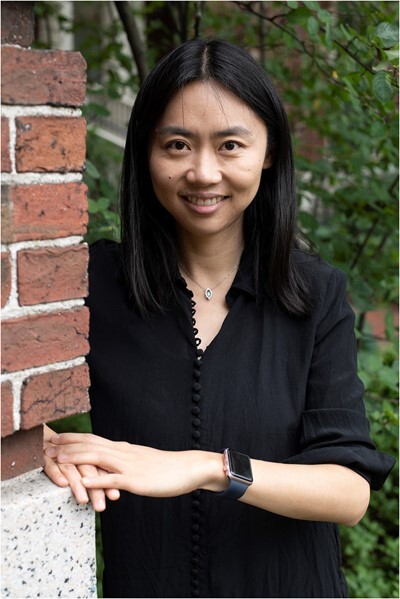
 *Jingyi Jessica Li, University of California, Los Angeles*

## Jingyi Jessica Li: at the junction of biology and statistics

Jingyi Jessica Li grew up in Chongqing, China, immersed in mathematics. Both of her parents were math majors and went on to become math teachers. Her mother in particular fostered Li’s mathematical curiosity, as she believed that everyone can learn and grow in mathematical understanding. She said, “My mom thought that math is like exercising. Everyone should do some exercise, even though we are not all top athletes going to the Olympics.” Although Li was exposed to math at a young age, she considered it a mature field and wanted to pursue studies in an area that could feed her curiosity. She entered Tsinghua University in China in 2003 and pursued a degree in biology, which was stoked by her interest in the Human Genome Project. She recalled, “It was a very exciting period with all these new technologies that could discover unknown things. I knew that to analyze this data we would need math, so I thought I should use my skills to approach biological questions. That’s why I decided to learn more statistics.”

Li pursued her interests in biology and statistics through her Ph.D. studies at the University of California, Berkeley, under the joint mentorship of Professors Peter J. Bickel and Haiyan Huang. Bickel is a world-renowned theoretical statistician and Huang is a statistician with expertise in bioinformatics. Bickel and Huang collaborated on bioinformatics projects, which offered Li the benefits of observing and learning from different perspectives in tackling research questions. When she joined their teams, they were both involved in the Encyclopedia of DNA Elements (ENCODE), which was developed as follow-up to the Human Genome Project to identify functional elements of the human genome. These studies generated enormous amounts of data due to the emergence of next-generation sequencing (NGS), leading to technologies including ChIP-seq [combining chromatin immunoprecipitation (ChIP) with NGS] and RNA-seq (using NGS to reveal the presence and quantity of RNAs). Li was interested in how to convert this type of raw data into numbers. She said, “We had not encountered this kind of data in statistics. How do we formulate this information into statistical questions? Sequence data are not numbers, so that forces the question to be important. It was very fun but challenging because we had to gain consensus on how to analyze those data. Everything was open and new.” She also recalled that statistics was a more rigid field set in dogma and theorems. Bioinformatics was more open and flexible, and she could use different approaches, such as computer algorithms or statistical models, so long as a biologically interesting question was being addressed.

Li’s fruitful Ph.D. research honed her skills to identify important bioinformatics problems and provide rigorous statistical solutions. Her work was published in high-profile journals and resulted in a faculty position in 2013 in the Department of Statistics, with a joint affiliation in the Department of Human Genetics, at UCLA. She was embraced by her new colleagues who mentored her through her first grant proposals, which yielded funding of an NIH RO1 grant on her first attempt. She was also the recipient of an NSF CAREER Award and Sloan Research Fellowship, serving as further recognition of her research contributions and potential as an independent investigator. Li attributes the early success of her young lab to her first graduate student, Wei Vivian Li, who is currently an Assistant Professor in the Department of Statistics at the University of California, Riverside. She considered working with (Vivian) Li like a mutual learning process as she was a new PI with a new Ph.D. student. They worked together through the early stages of establishing a research program, including gaining funding, and publishing papers. The success of this relationship set a very high benchmark for future graduate students in Li’s lab and helped Li grow as a mentor. She considers the most critical elements of successful mentorship to be transparency, open communication, finding a suitable project for a trainee, and pairing up students to encourage collaboration and mutual support.

Li’s intellectual curiosity has brought about her interest in improving the statistical rigor of genomic data analysis. She has had a long-standing interest in this area, and as a PI, she has more experience in developing more rigorous and computationally efficient and transparent solutions. One area where she has improved rigor is the control of false discovery rates (FDRs) in the differential expression (DE) analysis using RNA-seq data, in which a gene’s expression levels measured by RNA-seq are compared between two conditions, and the genes found as differentially expressed are “discoveries” of potential biological interests. Traditional DE analysis assigns a *P*-value to every gene by assuming every gene’s expression levels follow a negative binomial distribution under each condition. However, this assumption has not held up well when the RNA-seq samples under each condition are not experimental replicates, leading to invalid *P*-values and an inflated FDR—a co-discovery Li and her postdoc Dr. Xinzhou Ge made in a collaboration with Dr. Wei Li and his postdoc Dr. Yumei Li at UC Irvine. Li was inspired to look at the DE analysis in a different way after she heard a talk by a renowned Stanford statistician Professor Emmanuel Candes who developed the “knockoff filter” to control for FDRs when performing variable selection. This ultimately led Li and her team to develop the Clipper, which is a *P*-value-free FDR control method that is generally applicable to high-throughput data (such as NGS data) analysis, including the DE analysis.

Li is deeply involved in serving the fields of bioinformatics and statistics in many capacities, including work as a journal reviewer and editor, grant reviewer, and meeting organizer. She has developed both undergraduate and graduate courses featuring the use of statistics in computational biology, and her use of statistics to quantitate the Central Dogma is so widely recognized that it has been incorporated into the undergraduate textbook *Molecular Cell Biology*. Li is currently a Helen Putman Fellow at the Harvard Radcliffe Institute writing a statistical methods textbook focused on the selection of methods that are seemingly similar but have fundamental differences. She hopes that this book will be a useful tool to genomics researchers as they develop bioinformatics tools. She is also working on a statistical framework to address the evergreen question of whether cells belong to a single continuous trajectory or are discrete types.

Li’s publication record is diverse and highly cited, highlighting her strong record of outstanding interdisciplinary research at the nexus of statistics and biology. Her many awards and grants from the NIH, NSF, and other institutions highlight her visionary research, but she is truly grateful for the Overton Prize as it comes from her peers who also work at this unique juncture of science.

